# TMP- SSurface2: A Novel Deep Learning-Based Surface Accessibility Predictor for Transmembrane Protein Sequence

**DOI:** 10.3389/fgene.2021.656140

**Published:** 2021-03-15

**Authors:** Zhe Liu, Yingli Gong, Yuanzhao Guo, Xiao Zhang, Chang Lu, Li Zhang, Han Wang

**Affiliations:** ^1^School of Computer Science and Engineering, Changchun University of Technology, Changchun, China; ^2^School of Information Science and Technology, Institute of Computational Biology, Northeast Normal University, Changchun, China; ^3^Shanghai Mental Health Center, Shanghai Jiao Tong University School of Medicine, School of Biomedical Engineering, Shanghai Jiao Tong University, Shanghai, China; ^4^College of Intelligence and Computing, Tianjin University, Tianjin, China; ^5^College of Computing and Software Engineering, Kennesaw State University, Kennesaw, GA, United States

**Keywords:** transmembrane protein, deep learning, relative accessible surface area, attention mechanism, long short term memory

## Abstract

Transmembrane protein (TMP) is an important type of membrane protein that is involved in various biological membranes related biological processes. As major drug targets, TMPs’ surfaces are highly concerned to form the structural biases of their material-bindings for drugs or other biological molecules. However, the quantity of determinate TMP structures is still far less than the requirements, while artificial intelligence technologies provide a promising approach to accurately identify the TMP surfaces, merely depending on their sequences without any feature-engineering. For this purpose, we present an updated TMP surface residue predictor TMP-SSurface2 which achieved an even higher prediction accuracy compared to our previous version. The method uses an attention-enhanced Bidirectional Long Short Term Memory (BiLSTM) network, benefiting from its efficient learning capability, some useful latent information is abstracted from protein sequences, thus improving the Pearson correlation coefficients (CC) value performance of the old version from 0.58 to 0.66 on an independent test dataset. The results demonstrate that TMP-SSurface2 is efficient in predicting the surface of transmembrane proteins, representing new progress in transmembrane protein structure modeling based on primary sequences. TMP-SSurface2 is freely accessible at https://github.com/NENUBioCompute/TMP-SSurface-2.0.

## Introduction

Transmembrane Proteins (TMPs) are the gatekeepers to the cells and control the flow of molecules and information across the membrane (Goddard et al., 2015). The function of MPs is crucial for a wide range of physiological processes like signal transduction, electron transfer, and neurotransmitter transport (Roy, 2015). They span the entire biological membrane with segments exposed on both the outside and inside of aqueous spaces and have a profound effect on the pharmacokinetics of various drugs (Padmanabhan, 2014), cell mechanics regulation (Stillwell, 2016), molecule transport (Oguro and Imaoka, 2019; Puder et al., 2019) and so on. Also, the evidence is pointing toward TMPs associating with a wide range of diseases, including dyslipidemia, autism, epilepsy (Rafi et al., 2019; Tanabe et al., 2019; Weihong et al., 2019), and multiple cancers (Moon et al., 2019; Yan et al., 2019). Moreover, based on the current therapeutics market, it is evaluated that more than one-third of future drug targets would be TMPs (Studer et al., 2014) and the surface of TMPs is always identified as an interaction interface according to statistical reports (Lu et al., 2019b).

The quantitative approach for measuring the exposure of residues is to calculate the relatively accessible surface area (rASA) of the residues (Tarafder et al., 2018). rASA reflects the exposure of a single residue to the solvent, making it a directive reference of protein structures. Predicting rASA of TMPs is a rewarding task to biological problems like function annotation, structural modeling, and drug discovery (Zhang et al., 2019). In this case, accurate sequence-based computational rASA predictors need to be developed urgently to provide more support for structure prediction.

Many rASA predictors had been reported performing well on soluble proteins but the structural differences between the two protein types are significant, especially when interacting with the phospholipid bilayer. There are a few methods released to predict rASA of TMP residues based on their primary sequences. Beuming and Weinstein (2004) firstly proposed a knowledge-based method to predict the binary state (buried or exposed) of residues in terms of a preassigned cutoff in the transmembrane region of α-TMPs, it is the first rASA predictor of TMPs. After that, a series of methods using machine learning including SVC, SVR, and SVM emerged, which can be automatically divided into two categories according to their functionality: binary classifier and rASA real value predictor. All of these machine learning-based methods were designed for α-TMPs, some methods were just effective with the transmembrane region of the proteins restrictedly, such as TMX (Liwicki et al., 2007; Wang et al., 2011), TMexpoSVC (Lai et al., 2013), and TMexpoSVR (Lai et al., 2013), only MPRAP (Illergård et al., 2010) and MemBrane-Rasa (Xiao and Shen, 2015; Yin et al., 2018) were able to predict rASA of the entire sequence. Our previous work (Lu et al., 2019a) combined Inception blocks with CapsNet, proving that deep learning takes many advantages for the prediction but there is still room for accuracy improvement.

The predictors mentioned above including our previous version all applied common methods like SVM and feed-forward neural networks. However, these non-sequential models do not naturally handle sequential data and have trouble capturing long-term dependencies of a certain sequence (Sønderby and Winther, 2014), thus being a bottleneck in rASA prediction tasks, calling for more suitable models. In recent years, various Long Short Term Memory (LSTM) models have already employed to learn temporal information of protein secondary structure, confirming the amazing ability of LSTM in handling protein sequences through experimental verification (Sønderby and Winther, 2014; Sønderby et al., 2015; Heffernan et al., 2017). When it comes to sequence level issues, LSTM is definitely a better choice. Furthermore, previous tools did not have measures for reinforcing effective features, resulting in lower inefficiency of model learning. Additionally, various input restrictions and long waiting times also made the predictors less friendly to users.

In this study, we proposed an attention-enhanced bidirectional LSTM network named TMP-SSurface2 to predict rASA of TMPs at the residue level, which was implemented on top of the CNN-based Z-coordinate predictor TM-ZC (Lu et al., 2020). TMP-SSurface2 was trained and tested against the non-redundant benchmark dataset we created with primary sequences as input, improving the Pearson correlation coefficients (CC) value performance of the old version from 0.584 to 0.659, and reduced the mean absolute error (MAE) from 0.144 to 0.140. Apart from state-of-the-art prediction accuracy, TMP-SSurface2 also achieved the highest output efficiency compared to existing methods with no length restriction of input. The source codes of TMP-SSurface2 and the corresponding materials can be freely accessed at https://github.com/NENUBioCompute/TMP-SSurface-2.0.

## Materials and Methods

### Benchmark Dataset

A total of 4,007 TMPs were downloaded from PDBTM (version: 2019-01-04). We removed the proteins which contained unknown residues such as “X” or whose length was less than 30 residues since too short a sequence may not form a representative structure. To avoid the redundancy of data and reduce the influence of homology bias, CD-HIT (Li and Godzik, 2006) was utilized to eliminate the duplicate structures with a 30% sequence identity cut-off resulting in 704 protein chains (618 α protein chains and 86 β protein chains) left. These proteins were randomly divided into a training set of 604 proteins, a validation set of 50 proteins, and a test set of 50 proteins, respectively. In this work, five-fold cross-validation experiments were performed and the results were compared against other predictors.

The residue solvent accessibility surface area (ASA) is defined as the surface accessibility of a certain residue when exposed to water or lipid. Several tools are capable of calculating ASA, such as Naccess (Lee and Richards, 1971), PSAIA (Mihel et al., 2008), MSMS (Sanner et al., 1996), and Dictionary of Protein Secondary Structure (DSSP) (Kabsch and Sander, 1983).

The ASA of residues was calculating by DSSP, using a probe with a radius of 1.4 Å. A residue’s ASA is divided by the corresponding standard maximum accessible surface area (MaxASA), which is the ASA of extended tri-peptides (Gly-X-Gly) (Tien et al., 2013), to generate rASA values. rASA can be calculated by the following formula:

(1) $$r\,A\,S\,A=\frac{A\,S\,A}{M\,a\,x\,A\,S\,A}$$

### Features and Encoding

To make the prediction more accurate, it is vital to provide useful features to deep learning-based methods. In our experiments, we carefully select two encoding features to represent the protein fragment: one-hot code and PSSM.

Prediction of transmembrane protein residues’ rASA is a classical regression problem, which can be formulated as follows: for a given primary sequence of a TMP, a sliding window of *k* residues was used to predict the real value of central residue’s rASA. For instance, if *k* is 19, then each protein is subsequently sliced into fragments of 19 amino acids.

**FIGURE 1:**
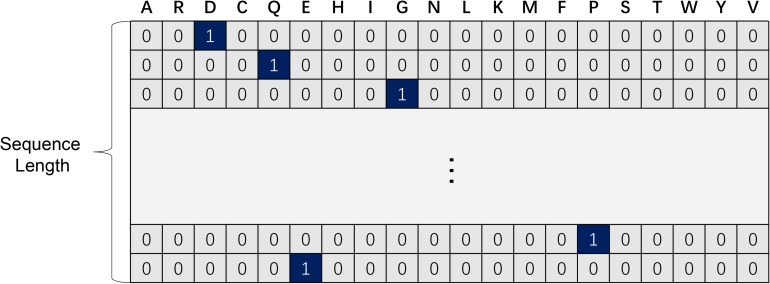
One-hot code of protein residues.

For each residue in protein sequences, one-hot code is a 20-dimension vector (see Figure 1), using a 19 dimensional “0” vector with a “1” corresponding to the amino acid at the index of a certain protein sequence. In this way, each protein fragment can be mapped into an exclusive and undisturbed coding within its relative position information (He et al., 2018). It is proved that a one-hot code is extremely easy to generate while effective for protein function prediction associated problems (Ding and Li, 2015).

A position-specific scoring matrix (PSSM) reflects the evolutionary profile of the protein sequence based on a search against a certain database. Highly conserved regions during evolution are always functional regions according to the researches (Jeong et al., 2010; Zeng et al., 2019), so PSSM has been widely used in many bioinformatics problems and achieves commendable results. In our study, PSI-BLAST (Altschul et al., 1997) was utilized to generate PSSM searching against the uniref50 (version: 2019-01-16) database with 3 iterations and a 0.01 *E*-value cutoff. For a given protein sequence, the PSSM feature is a 20-dimension matrix with each column representing a profile and each row representing a residue.

**FIGURE 2:**
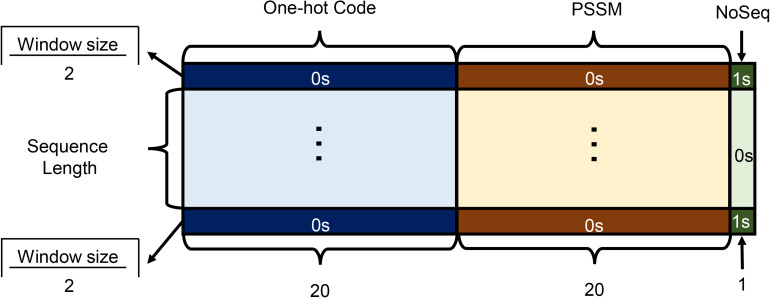
Encoding features as the model input.

As shown in Figure 2, each amino acid in the protein sequence is represented as a vector of 41 numbers, including 20 from one-hot code (represented as binary numbers), 20 from PSSM, and 1 Noseq label (representing a gap) (Fang et al., 2018) in the last column to improve the prediction performance of the residues located on both ends of protein while using a sliding window. In order to facilitate the window sliding operation, the first and last parts of the sequence are, respectively, padded with 1 and 0 s, which length is half of the sliding windows size. For each protein with L residues, we can get L matrices.

### Model Design

**FIGURE 3:**
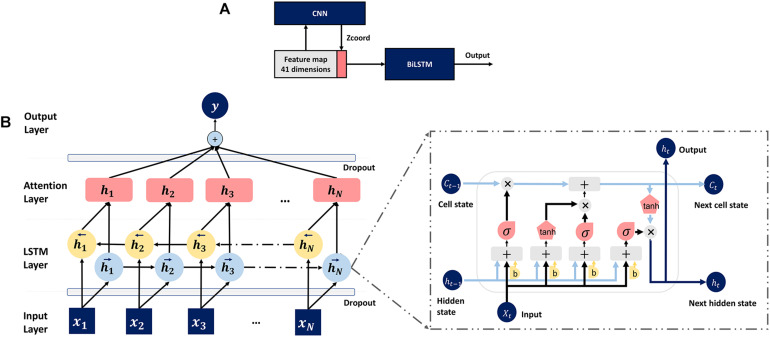
**(A)** Pipeline of the deep learning model. **(B)** The attention-enhanced bidirectional LSTM network.

In this section, a novel compound deep learning network is presented. Figure 3A shows the proposed pipeline. The input features for TMP-SSurface2 are the one-hot code and the PSSM matrix. The CNN whose structure and parameters are all same as TM-ZC is used to generate the Z-coordinate of TMP residues. Z-coordinate, which is an important constituent in the field of MP structure prediction, is often implemented to stand for a residue’s relative position concerning the membrane (Yin et al., 2018). After that, the final feature map containing a one-hot code, PSSM, and Z-coordinate will be put into a bidirectional LSTM (BiLSTM) network for training and testing.

To further optimize the model, we also attached an attention mechanism (Baron-Cohen, 1995) layer to the top of BiLSTM, which is motivated by how we pay visual attention to different regions of an image or correlate words in one sentence, to help LSTM focus on a certain region that relatively deserves more attention. The detailed structure of the mentioned LSTM network is shown in Figure 3B.

Formula (2) to formula (9) describe the forward recursions for a single LSTM layer, where ⊙ equals to the elementwise multiplication, *x_t* means input from the previous layer,*i*_*t*_,*f*_*t*_, *o_t* represent “input gate,” “forget gate” and “output gate,” respectively.*h*_*t*−*rec*_ stands for the output forwarded to the next time slice, and *h_t* is passed upwards in a multilayer LSTM (Sønderby and Winther, 2014). Attention neural networks have recently demonstrated popularity in a wide range of tasks ranging from natural language processing to computer vision (Chorowski et al., 2014; Rocktäschel et al., 2015; Sharma et al., 2015). Inspired by these projects, we attached an attention mechanism to LSTM for feature capturing. As shown in formula (10), the combination of attention mechanism enables the model to re-assign the weight (*W*_*att*_) of the feature vector (*V*), indicating that the next output vector (*V*′) should focus more on which part of the input sequence, and then generate the next output according to the focus region.

(2) $$i_{t}=\sigma \,\left(x_{t}\,W_{x_{i}}+h_{t-1}\,W_{h_{i}}+b_{i}\right)$$

(3) $$f_{t}=\sigma \,\left(x_{t}\,W_{x\,f}+h_{t-1}\,W_{h\,f}+b_{f}\right)$$

(4) $$o_{t}=\sigma \,\left(x_{t}\,W\,x_{o}+h_{t-1}\,W_{h_{0}}+b_{o}\right)$$

(5) $$g_{t}=t\,a\,n\,h\,\left(x_{t}\,W_{x\,g}+h_{t-1}\,W_{h\,g}+b_{g}\right)$$

(6) $$c_{t}=f_{t}\,⊙c_{t-1}+i_{t}\,⊙g_{t}$$

(7) $$h_{t}=o_{t}\,⊙t\,a\,n\,h\,\left(c_{t}\right)$$

(8) $$h_{t-r\,e\,c}=h_{t}+f\,e\,e\,d\,f\,o\,r\,w\,a\,r\,d\,n\,e\,t\,\left(h_{t}\right)$$

(9) $$\sigma \,\left(z\right)=\frac{1}{1+e\,x\,p\,\left(-z\right)}$$

(10) $$V^{\prime }=W_{a\,t\,t}\,⊙V$$

Our model was implemented, trained, and tested using Keras and Tensorflow. Main hyperparameters (sliding window size, training dropout rate, number of LSTM units, and layers of LSTM) were explored. The early stopping and save-best strategy were applied when the validation loss did not reduce in 10 epochs during training time, the process would stop and save the best model parameters. We used Adam optimizer to dynamically transform the learning rate while the model was training. All the experiments were performed using an Nvidia 1080Ti GPU.

### Performance Evaluation

To quantitatively evaluate the predictions of TMP-SSurface2, Pearson correlation coefficients (CC) and mean absolute error (MAE) were used in this study. CC undertook the task of measuring the linear correlation between real values and predicting values. CC ranges from −1 to 1, where −1 indicates an abstract negative correlation, 1 positive correlation, and 0 absolutely no correlation. Formula (11) shows the definition of CC, where L represents the number of residues, *x_i* and *y_i* define the observed and predicted rASA value severally, $\bar{x}$ and $\bar{y}$ equal to the corresponding mean value, respectively.

(11) $$C\,C=\frac{\sum_{i=1}^{L}\left(x_{i}-\bar{x}\right)\,\left(y_{i}-\bar{y}\right)}{\sqrt{\left[\sum_{i=1}^{L}{\left(x_{i}-\bar{x}\right)}^{2}\right]\,\left[\sum_{i=1}^{L}{\left(y_{i}-\bar{y}\right)}^{2}\right]}}$$

Mean absolute error measures the closeness of prediction values to real values. As shown in formula (12), MAE is defined as the average difference between predicted and observed rASA values of all residues.

(12) $$M\,A\,E=\frac{1}{L}\,\sum_{i=1}^{L}\left|y_{i}-x_{i}\right|$$

## Results

### Feature Analysis

As we all know, it is the features, instead of model structures, determine the upper-performance limit of deep learning. To investigate the different features’ contribution to the predictor TMP-SSurface2, we tested both independent features used in the predictor and their various combinations on our valid dataset.

Table 1 illustrates that all of the three independent features (Z-coordinate, one-hot, and PSSM) contain useful information for predicting rASA by themselves, among which PSSM achieves the best overall results (CC = 0.631 and MAE = 0.144). It is suggested that PSSM is an important feature in rASA prediction mainly because of the inclusion of evolutionary knowledge. When combining these different features, as was indicated by a former study, the CC values are almost linearly related to the MAE values (Yuan et al., 2006), the maximum CC values always accompany the minimum MAE. Experimental investigation shows that every single feature made a contribution to the prediction and achieved the most considerable performance (CC = 0.659 and MAE = 0.140) when they were combined.

**TABLE 1:** Prediction performance based on individual input features and their various combinations.

Feature	CC	MAE
Z-coordinate	0.310	0.191
one-hot	0.417	0.180
PSSM	0.631	0.144
one-hot+PSSM	0.641	0.142
one-hot+PSSM+ Z-coordinate	**0.659**	**0.140**

*Bold fonts represent the best experimental results.

### Hyperparameter Tuning and Model Performance

Tables 2–5 summarizes the exploration of the attention-enhanced bidirectional LSTM network with various hyperparameters on the validation dataset. The object of doing these experiments was to find out a better configuration of our method. The tested hyperparameters were carefully selected and only the major factors which would greatly influence the model were explored on the validation dataset.

**TABLE 2:** Effect of sliding window length on CC performance.

Window Length	CC	MAE
13	0.642	0.141
15	0.641	0.143
17	0.645	0.143
19	**0.648**	**0.140**
21	0.646	0.141
23	0.640	0.142

*Bold fonts represent the best experimental results.

**TABLE 3:** Effect of dropout rate on CC performance.

Dropout rate	Train CC	Test CC	Test MAE
No	0.851	0.632	0.143
0.2	0.806	0.640	0.143
0.3	**0.782**	**0.648**	**0.140**
0.4	0.762	0.641	0.141
0.5	0.725	0.638	0.143

*Bold fonts represent the best experimental results.

**TABLE 4:** Effect of LSTM units’ number on CC performance.

Num of units	CC	MAE	Num of Parameters
500	0.639	0.142	2,191,381
600	0.641	0.142	3,109,591
700	**0.648**	**0.140**	**4,187,781**
800	0.643	0.143	5,425,981
900	0.646	0.140	6,824,181

*Bold fonts represent the best experimental results.

**TABLE 5:** Effect of the number of LSTM layers on CC performance.

LSTM Layers	CC	MAE	Num of parameters
1	0.648	0.140	4,187,781
2	**0.659**	**0.140**	**15,953,381**
3	0.642	0.141	27,718,981
4	0.646	0.141	39,484,581

*Bold fonts represent the best experimental results.

A sliding window approach is utilized to append useful neighborhood information to improve prediction accuracy. Table 2 shows how the length of the sliding window affects the performance of our network. Since the contexts fed into the proposed deep learning model relies on the length of the sliding window, the prediction accuracy would be directly influenced by its value. In general, when the window size becoming larger, it will cost more time for training, but the prediction performance may not be better as the window length increases. Historically, if a sliding window was utilized by sequence-based protein structure predicting tasks, the peak of performance often occurred when its length was between about 13 and 23 residues (Fang et al., 2018; Lu et al., 2019a). We searched the window length from 13 to 23 by a step of two residues, finding the best result when the number is 19 and it was chosen as the final window length in this section.

Table 3 shows how the dropout rate affects the model performance when the window size is 19. Deep learning neural networks are much easier to overfit a training dataset with few examples, dropout regularization will help reducing overfitting and improve the generalization of deep neural networks (Dahl et al., 2013). The dropout rates in the range of 0.2–0.4 are all acceptable according to the training and testing prediction performance. Finally, we chose 0.3 as our dropout rate, and the concatenation network in our study is regularized using a 30% dropout.

In the LSTM network, the number of LSTM units is also an important parameter, which determines the output dimension of different layers just like ordinary neural networks. When the number of LSTM units in one layer changes, the scale of parameters and prediction accuracy of the model will immediately be affected. To find the best choice of LSTM units, we tried different values at the same time. The results are shown in Table 4, we chose 700 as the number of LSTM units in a simple layer.

As it can be seen in Table 5, when the LSTM network has two bidirectional layers (i.e., four simple layers, two forward and two backward), the model performs best on the validation set. However, the prediction accuracy of the model may not grow as the number of LSTM layers increases. It is suspected that a large number of model parameters will lead to the overfitting of LSTM on the training set, thus reducing the generalization ability of it.

### Comparison With Previous Predictors

In this section, we list the existing methods that can be used to predict the rASA of TMP in the full chain and compare TMP-SSurface2 with them. Table 6 shows the performance improvement of the proposed TMP-SSurface2 after implementing the new model relative to the old version and the other tools. During testing MPRAP and MemBrane-Rasa on the independent dataset, we figured out that not every sequence fed into these predictors can get a corresponding output since some third-party tools might cause the failure. Just like TMP-SSurface, the new version is reliable in getting prediction results because of the simple coding scheme. Furthermore, TMP-SSurface2 significantly outperformed the previous predictors and has the quickest predicting speed. The details of the comparison are shown in Table 6.

**TABLE 6:** Comparison of TMP-SSurface2 with the previous predictors on the independent dataset.

Predictor	CC	MAE	Failure	Time Cost (min)
MPRAP	0.397	0.176	9	6.5
MemBrane-Rasa	0.545	0.153	7	23.7
TMP-SSurface	0.584	0.144	0	4.7
TMP-SSurface2	**0.659**	**0.140**	**0**	**4.3**

*Bold fonts represent the best experimental results.

### TMP Type Test

Statistical results show that most of the existing methods only focused on α-helical TMPs while ignored β-barrel TMPs, which made it inconvenient for the users who cannot distinguish the protein type. As described previously, the data set we used contains both α-helical and β-barrel TMPs, making our predictor more suitable for all types of TMP. Table 7 illustrates that when TMP-SSurface2 meets either of these two different TMPs, the prediction performance on the independent testing dataset was both considerable and reliable.

**TABLE 7:** Performance of TMP-SSurface2 on different types of TMPs.

TMP Types	Protein number	CC	MAE
α-helical TMPs	45	0.674	0.138
β-barrel TMPs	5	0.562	0.151
all-TMPs	50	0.659	0.140

### Contribution of Attention Mechanism

**FIGURE 4:**
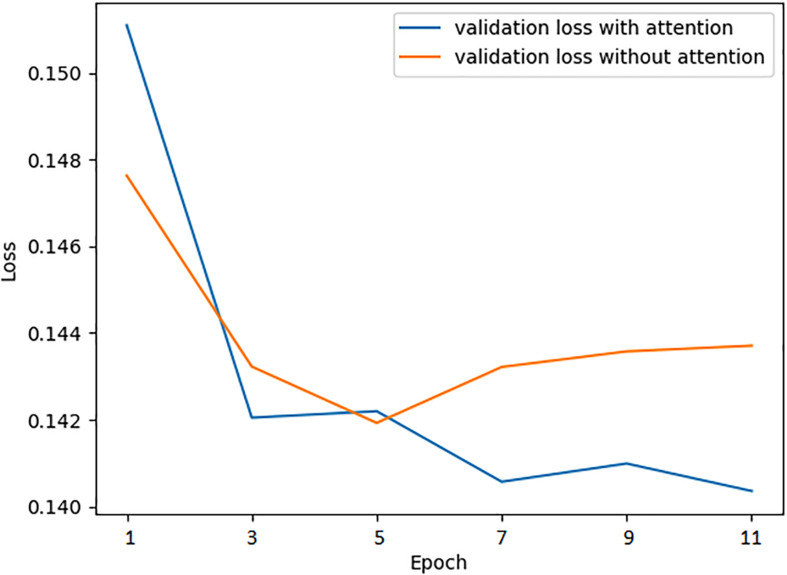
Validation loss curve of the training process with and without attention mechanism.

The attention mechanism promotes the model to extract features more effectively, speeding up the prediction accuracy to the peak, even improving the performance at the same time. To verify the positive effect of the attention mechanism, we monitoring the mean absolute error loss curve of the validation dataset with or without the attention layer, respectively, using the preselected best hyperparameters while training. As is shown in Figure 4, when the network is attention-enhanced, the convergence speed and accuracy of the training set were significantly improved.

Moreover, we also combined attention mechanisms with various network layers to verify whether or how much the attention mechanism would improve the prediction performance. Firstly, we removed the attention layer and tested the trained model on the test set. Meanwhile, we attached the attention mechanism to the bidirectional LSTM layer and the Dropout layer, respectively, to conduct experiments, the results are shown in Table 8. It can be seen that the combination of attention mechanism and bidirectional LSTM layer reached the best performance, which is related to the fact that the LSTM layer had learned the most abundant features. In essence, the attention mechanism is to enhance the feature extraction process, so it will achieve the best effect when combined with the network layer that is the most effective for feature extraction.

**TABLE 8:** Contribution of attention mechanism.

Model	CC	MAE
No attention	0.637	0.150
Attention with LSTM	**0.659**	**0.140**
Attention with Dropout	0.645	0.141

*Bold fonts represent the best experimental results.

### Visualization of the Features Learnt by LSTM

**FIGURE 5:**
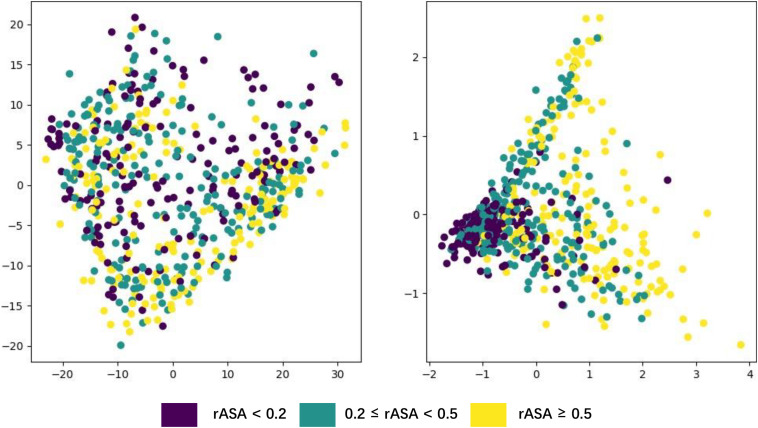
Visualization of the features learned by LSTM using PCA.

Deep neural networks can learn high-level abstract features from original inputs, to verify whether the extracted features are generalizable, we utilized PCA (Wold, 1987) to visualize the input features and each LSTM unit’s output in one bidirectional layer with test data. Figure 5 shows the PCA scatter diagram of the test data before and after fed into LSTM, respectively. The input data had 42 features (i.e., 42 dimensions), PCA reduced its dimensionality and visualized it, but there was no clear cluster. The bidirectional LSTM layer we used contained 1,400 dimensions (twice of units in a simple LSTM layer) and the trend toward clustering had occurred, which demonstrates that LSTM had effectively captured useful and powerful features needed in this work.

Generally, buried residues are under stronger evolutionary constraints than exposed ones irrespectively of the environment (Kauko et al., 2008). The diagram shows that the residues whose rASA was lower than 0.2 narrowed down to a small area through PCA, which means these residues’ rASA values stayed closely aligned with the features derived from their sequence, just proved the previous statement.

### Case Studies

**FIGURE 6:**
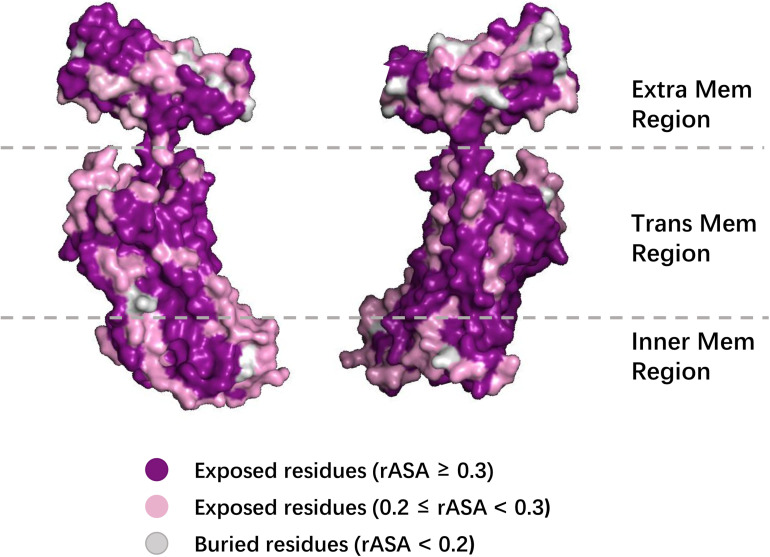
The 3D visualization of the predicted result (surface version).

**FIGURE 7:**
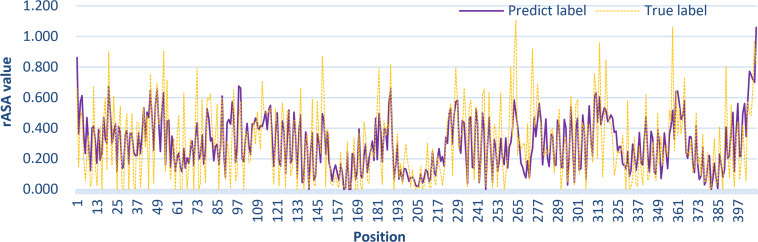
The comparison between the TMP-SSurface2-predicted rASA values and real rASA values.

To further demonstrate the effectiveness of TMP-SSurface2, we take 4n6h_A as an example of case studies. 4n6h_A is an Escherichia coli α-TMP (subgroup: G protein-coupled receptor) containing 408 residues as the receptor of multiple ligands like sodium ion, heme, and so on (Fenalti et al., 2014). Figure 6 shows the 3D visualization of the predicted result (surface version) and Figure 7 illustrates the comparison between the TMP-SSurface2-predicted rASA values and real rASA values. As were shown in figures, the overall trend of rASA has been appropriately captured, but TMP-SSurface2 seems conservative in predicting some fully exposed or buried residues’ rASA. It is suspected that TMP-SSurface2 may confuse these residues with the ones located on water-soluble regions, resulting in low prediction performance of them.

## Conclusion

In this study, we proposed an updated TMP-SSurface predictor, which aimed to predict transmembrane protein residues’ rASA from primary sequences. Apart from classical feed-forward neural networks, we developed an attention-enhanced bidirectional LSTM network on top of the CNN-based Z-coordinate predictor to process sequential data and improved the CC value performance of the old version from 0.58 to 0.66 on the independent test dataset. The improvement of LSTM directly indicates that the order of residues in a sequence would exactly influence the protein structure and LSTM has a more powerful ability to process sequential data than CapsNet. The Z-coordinate feature was explored and applied in TMP-SSurface2 and proved to be useful, which means the z-coordinate has a lifting effect on rASA prediction, indicating that structural features can support each other. We also appended various important experiments like feature visualization and case study to visualize the effectiveness of the model. TMP-SSurface2 had no constraints with input since it could handle all types of TMPs at any length. The predicted rASA would make contributions to TMPs’ structure analysis, TMP-ligand binding prediction, TMP function identification and so on.

## Data Availability Statement

The original contributions presented in the study are included in the article/supplementary material, further inquiries can be directed to the corresponding author.

## Author Contributions

ZL, YGo, and XZ conceived the idea of this research, collected the data, implemented the predictor, and wrote the manuscript. YGu and CL tuned the model and tested the predictor. LZ and HW supervised the research and reviewed the manuscript. All authors contributed to the article and approved the submitted version.

## Conflict of Interest

The authors declare that the research was conducted in the absence of any commercial or financial relationships that could be construed as a potential conflict of interest.
